# Identifying cholera "hotspots" in Uganda: An analysis of cholera surveillance data from 2011 to 2016

**DOI:** 10.1371/journal.pntd.0006118

**Published:** 2017-12-28

**Authors:** Godfrey Bwire, Mohammad Ali, David A. Sack, Anne Nakinsige, Martha Naigaga, Amanda K. Debes, Moise C. Ngwa, W. Abdullah Brooks, Christopher Garimoi Orach

**Affiliations:** 1 Department of Community Health, Uganda Ministry of Health, Kampala, Uganda; 2 Department of International Health, Johns Hopkins Bloomberg School of Public Health, Baltimore, Maryland, United States of America; 3 Department of National Disease Control, Uganda Ministry of Health, Kampala, Uganda; 4 Department of Environmental Health, Uganda Ministry of Water and Environment, Kampala, Uganda; 5 Department of Community and Behavioural Sciences, College of Health Sciences, Makerere University, Kampala, Uganda; Massachusetts General Hospital, UNITED STATES

## Abstract

**Background:**

Despite advance in science and technology for prevention, detection and treatment of cholera, this infectious disease remains a major public health problem in many countries in sub-Saharan Africa, Uganda inclusive. The aim of this study was to identify cholera hotspots in Uganda to guide the development of a roadmap for prevention, control and elimination of cholera in the country.

**Methodology/Principle findings:**

We obtained district level confirmed cholera outbreak data from 2011 to 2016 from the Ministry of Health, Uganda. Population and rainfall data were obtained from the Uganda Bureau of Statistics, and water, sanitation and hygiene data from the Ministry of Water and Environment. A spatial scan test was performed to identify the significantly high risk clusters. Cholera hotspots were defined as districts whose center fell within a significantly high risk cluster or where a significantly high risk cluster was completely superimposed onto a district. A zero-inflated negative binomial regression model was employed to identify the district level risk factors for cholera. In total 11,030 cases of cholera were reported during the 6-year period. 37(33%) of 112 districts reported cholera outbreaks in one of the six years, and 20 (18%) districts experienced cholera at least twice in those years. We identified 22 districts as high risk for cholera, of which 13 were near a border of Democratic Republic of Congo (DRC), while 9 districts were near a border of Kenya. The relative risk of having cholera inside the high-risk districts (hotspots) were 2 to 22 times higher than elsewhere in the country. In total, 7 million people were within cholera hotspots. The negative binomial component of the ZINB model shows people living near a lake or the Nile river were at increased risk for cholera (incidence rate ratio, IRR = 0.98, 95% CI: 0.97 to 0.99, p < .01); people living near the border of DRC/Kenya or higher incidence rate in the neighboring districts were increased risk for cholera in a district (IRR = 0.99, 95% CI: 0.98 to 1.00, p = .02 and IRR = 1.02, 95% CI: 1.01 to 1.03, p < .01, respectively). The zero inflated component of the ZINB model yielded shorter distance to Kenya or DRC border, higher incidence rate in the neighboring districts, and higher annual rainfall in the district were associated with the risk of having cholera in the district.

**Conclusions/significance:**

The study identified cholera hotspots during the period 2011–2016. The people located near the international borders, internationally shared lakes and river Nile were at higher risk for cholera outbreaks than elsewhere in the country. Targeting cholera interventions to these locations could prevent and ultimately eliminate cholera in Uganda.

## Author summary

Uganda has regularly reported cholera since its first appearance in 1971. Although the Government of Uganda implements cholera prevention and control interventions such as provision of safe water, promotion of sanitation and hygiene, health education and healthcare, the disease continues to threaten many districts in the country. The population with access to improved water supply in the urban and rural areas were 71% and 67% respectively, and with access to improved sanitation was 86% in urban areas and 79% in rural areas. Identifying the districts with increased risk is an important step in defining areas where additional preventive interventions are needed. We used district level confirmed cholera outbreak data for a six year period (2011–2016), and identified cholera “hotspot” districts. Rates of cholera in these districts, with a population of about 7 million, are 2 to 22 times higher than elsewhere in the country. These “hotspots” located along the international borders with Democratic Republic of Congo (DRC) and Kenya and the internationally shared lakes and river Nile. Targeted cholera prevention and control interventions to the hotspots in Uganda could lead to reduction in cholera cases and deaths. The hotspots identified herein provide an affordable way of implementation of the comprehensive cholera prevention and control mechanism as recommended by WHO. Since the hotspots are along the international borders, collaboration with the neighboring countries is the key to eliminate cholera in Uganda and the region as a whole.

## Introduction

Since 1970, cholera has become endemic in many countries of sub-Saharan Africa and remains a recurring problem [1]. During 2007 and 2011, annual Case-Fatality Ratios (CFRs) for cholera within this region ranged from 2.22% to 2.95%, and exceeded 5% in a country in each year [2–6]. It is evident that in spite of continued scientific advancement in prevention and treatment of cholera, it remains a major public health issue in many parts of sub-Saharan Africa.

Cholera incidence and mortality, and diarrheal diseases, can be reduced through increased access to safe water, sanitation and hygiene (WaSH) and through behavioral change resulting from education and training in communities [7] as was done in Latin America [8, 9] and in industrialized countries [10]. However, improvements in WaSH infrastructure have been slow in many cholera affected African countries [11, 12], resulting in repeated cholera outbreaks.

According to WHO, cholera prevention and control should be a priority in areas at risk for cholera, and vaccines should be included in conjunction with other cholera prevention and control strategies [13]. Safe WHO prequalified cholera vaccines (OCV) [14] provide 65% protection at least for 3 to 5 years [15]. Studies have also demonstrated that large-scale vaccination campaigns are feasible and acceptable, reflected by high acceptance rate and high coverage in both urban and rural settings [16–20]. To ensure access to OCV for cholera affected countries, a global stockpile is now available for both emergency use to control outbreaks and areas with recurrent cholera outbreaks. However, the supply of OCV is limited compared to the large number of people living in countries at risk [21]. Thus, countries with cholera need to identify specific areas at increased risk in order to focus their control efforts using an integrated approach with WaSH improvements and vaccination.

In Uganda, epidemics of cholera have occurred regularly since the disease first appeared in 1971 [22]. The Government of Uganda instituted preventive and control measures that included promotion of access to safe water, sanitation and hygiene; health education and community mobilization; disease surveillance; and case management [23]. However cholera cases continues to be reported annually [24]. It is important for the country to understand the extent of the problem, identify the areas of high risk, and use this information to plan for an effective intervention strategy. In most areas, during cholera outbreaks, cases tend to be clustered in specific areas and among certain population groups [25, 26], which can be demonstrated by the spatial epidemiology of the disease. A study that describes the spatial distribution of disease, its incidence using geographic information system (GIS) and its association to potential risk factors should help guide interventions to control cholera [27]. The aim of this study was to identify cholera high risk districts (hotspots) in Uganda in order to provide insights and guidance for prevention, control and ultimately elimination of cholera in Uganda.

## Material and methods

### The study area

Uganda, is divided into four geographical regions (Central, Eastern, Western, and Northern) with a projected total of 36.6 million people as of June 2016, and annual growth rate of 3.0 percent. There were 116 districts according to June 2016 data. However, in this study we used the 2014 census with 112 districts (S1 Fig). The country has tropical weather conditions moderated by the high altitudes. The Central, Eastern, and Western regions of the country have two rainy seasons per year, with heavy rains from March to early June and light rains between September and December. The level of rainfall decreases toward the north, with just one rainy season from April to October [27]. Uganda’s vegetation varies between tropical rain forest in the south and the savannah woodlands and semi-desert region in the northeast [28]. The population density is higher in the Central and Western regions and declines toward the North [29].

### Cholera data

District level confirmed cholera outbreak data from 2011 to 2016 were abstracted from the Uganda Ministry of Health, Health Management Information System disease surveillance database. In order for the districts to confirm cholera outbreaks, 10–20 stool samples are collected from suspected cholera cases and sent for culture to a laboratory in Kampala for isolation of *Vibrio cholerae* O1 and O139. If a sample is found positive then it is defined as a confirmed outbreak. The district health workers are guided by the following standardized case definition for cholera case detection and confirmation below [30]:

Suspected case:

- In a patient age 5 years or more, severe dehydration or death from acute watery diarrhea.
- If there is a cholera epidemic, any person age 2 years or more with acute watery diarrhea, with or without vomiting.

**C**onfirmed case:

- *Vibrio cholerae* O1 or O139 was isolated in the stool in a lab test.

In this study, we included only those cases for which the outbreaks were confirmed as per national standard guidelines for cholera prevention and control.

### Population and rainfall data

The population data were obtained from the Uganda Census 2014 posted in the website of the Ministry of Internal Affairs, Uganda Bureau of Statistics (UBOS) 2016 [31]. We used UBOS definition to categorize areas into urban and rural. According to UBOS, the City, Municipality, Town Council or Town Board are the as urban areas. In 2014, Uganda had 197 urban centers (one City, 22 Municipalities and 174 Town Councils) with a total population of 6 million urban areas. The size of the urban centers varied widely, from Kampala City with 1.5 million persons to small Town Councils with less than 5,000 persons.

We obtained annual rainfall data of 2011–2015 from the 2016 Statistical Abstract of the UBOS (http://www.ubos.org/onlinefiles/uploads/ubos/statistical_abstracts/2016%20Statistical%20Abstract.pdf). The data were available from 8 stations throughout the country. We linked the districts to its nearest station, and obtained the rainfall data for the period. We, then, calculated average annual rainfall for each of the districts.

### Water and sanitation data

The water, sanitation and hygiene data were obtained from the 2016 Uganda Ministry of Water and Environment Annual Report [32]. In this report, improved water supply sources included the boreholes, protected springs, shallow wells, and rainwater harvesting tanks. Improved piped water supply outlets included public stand posts, yard taps, kiosks, house (domestic) connections and institutional connections. Water for production facilities (dams and valley tanks) were not regarded as improved water supplies for domestic use. The population with access to improved water supply in the urban and rural areas were 71% and 67% respectively. The was achieved due to back-up support for operations and maintenance provided by regionally based Umbrella Organizations (Central Umbrella based in Wakiso; Mid-Western Umbrella based in Kyenjojo; South Western Umbrella based in Kabale; Northern Umbrella based in Lira; Eastern Umbrella based in Mbale; and Karamoja Umbrella based in Moroto) [33].

Improved sanitation (defined as not shared, cleanable, sealable and durable) was reported to be 86% in urban areas and 79% in rural areas [32]. However, most of the latrines did not meet the standards of the WHO/UNICEF Joint Monitoring Program (JMP), which estimated that only 35% of the rural people in Uganda had access to improved sanitation, with an estimated 10% practicing open defecation. The JMP also estimated that only 34% of the urban population has access to improved sanitation as half of the urban population uses improved but shared facilities. One percent of the urban population was estimated to practice open defecation, while 15% use unimproved facilities [33].

We also collected district level data on a sanitation and hygiene benchmark (the higher the benchmark score the better the sanitation and hygiene condition) from the same report [33]. This was calculated as the sum of scores of the following items:

1. average increase in household level sanitation coverage from 2011 to 2016 (≥3% = 10, 2% = 5, 1% = 3, <1% = 0)
2. cost of the toilet (Top 10 = 10, 11^th^ to 20^th^ = 7, 21^st^ to 30^th^ = 3, ≥31^st^ = 0)
3. pupils per latrine stance ratio (≤40 = 15, 41–50 = 10, 51–60 = 5, >61 = 0)
4. household sanitation coverage (>70% = 25, 50–69% = 20, 25–49% = 15, <24% = 0)
5. hand washing coverage (≥50% = 15, 23–49% = 10, 10–22% = 5, <9% = 0)
6. number of open defecation free (ODF) villages (≥51 = 10, 21 to 50 = 8, 1 to 20 = 5, Nil = 0)
7. percentage of triggered village that are ODF (≥51% = 15, 21% to 50% = 10, 1% to 20% = 5, 0% = 0).

### GIS data

The digital maps of Uganda were obtained from the Energy Sector GIS Working Group Uganda, which is an open data (http://data.energy-gis.opendata.arcgis.com/datasets/f0d63758fb8f4ded85394b51594d294a_0). The digital map of the health facilities in Uganda was abstracted from a free, open, collaborative platform for creation and maintenance of geocoded health facility master list (https://healthsites.io/). The other geographic features such as road, railway, and waterbodies were obtained from DIVA-GIS (http://www.diva-gis.org/Data), an open source platform. These maps were projected in WGS 1984 UTM zone 36S for conducting spatial analysis. We used ArcGIS 10.4.1 (ESRI Inc.) for mapping the cholera risk in the country and obtaining spatial variables such as distances to the nearest lake and the nearest health facility from the centroid of each of the districts. We also calculated population density (in km^2^) using total population in the district divided by the size of the area of the district.

### Spatial scan statistic to identify hotspots

We used SaTScan version 9.4.4 (http://www.satscan.org/) to identify cholera hotspots in Uganda. In particular, we employed the Poisson-based spatial scan statistic, because the cases in the districts follow a Poisson distribution. The population in the districts were adjusted in detecting the spatial clusters (hotspots). Under the Poisson model, we assumed the expected number of cases in each part of the study area is proportional to its population size. The model detected clusters in a multidimensional point process and allowed variable window sizes to scan for the cases within the study area. The variable window size was chosen, as we did not have prior knowledge about the size of the area covered by a cluster. We selected circular scan window, and the radius of the window was chosen to vary from 0% to 20% of the population at risk. We started with 0% due to use of the center coordinates of the districts as the geographic reference point. The clusters indicate areas with significantly higher rates inside the window compared to that outside the window. Since the location and size of the window were changed during the operation, it created an infinite number of distinct geographical circles. Therefore, computing the number of points at any given time was not possible [34], leading us to calculate the likelihood ratio. Under the Poisson model, the likelihood function for a specific window is:

$${\lambda =(\frac{n}{\mu })}^{n}{\left(\frac{N-n}{N-\mu }\right)}^{N-n}I\;(n>\mu )$$

where,

*N* = number of cases in the study area
*n* = number of cases within the window
*μ* = expected number of cases within the window under the null hypothesis
*I()* = an indicator function

Since we scanned for clusters with only the high rates, *I()* is 1 when the window had more cases than expected under the null hypothesis, and in all other cases it was 0. The likelihood function was maximized over all windows, identifying the window that constituted the most likely cluster. The most likely cluster (hotspots) is the area that is least likely to have occurred by chance. The likelihood ratio for the window was noted and constituted the maximum likelihood ratio test statistic. Its distribution under the null hypothesis and its corresponding p-value were determined by repeating the same procedure on a large number of random replications of the data set generated under the null hypothesis using a Monte Carlo simulation approach.

### Spatial variables

We calculated distance (in kilometer) from the centroid of the district to the nearest hospital (in linear distance), nearest DRC or Kenya border, and the nearest bank of lake or river. To calculate weighted incidence rate of the 1^st^ order of neighbors for each district, we first created a spatial weight matrix for each district considering 1^st^ order of neighbor districts using a GIS tool and then calculated weighted incidence rate of the neighbor districts using cholera cases over the six year period and the population in those districts. Note that we chose 1^st^ order of neighbor districts, because we believe that cholera transmission would less likely to go beyond the 1^st^ order of neighbor district.

### Statistical analysis to identify the risk factors

To evaluate whether the risk for cholera is associated with water and sanitation conditions as well as other spatial characteristics of the districts, we used the zero-inflated negative binomial (ZINB) model. The ZINB model was chosen because our data were a two-component mixture composed of at-risk districts whose responses (i.e. cholera cases) follow Poisson process with overly dispersed (variance exceeds the mean), i.e., negative binomial (NB) and non-risk districts whose responses are constant, i.e., zero [35]. With probability π, the response of the first process is a zero count, and with probability of (1 − π) the response of the second process is governed by a NB with mean λ, which also generates zero counts. The overall probability of zero counts is the combined probability of zeros from the two processes. Thus, a ZINB model for the response Y can be written as:

$$P\left(Y=0\right)=\pi +(1-\pi ){\left(1+k\lambda \right)}^{-1/k}$$

$$P\left(Y=y\right)=(1-\pi )\frac{\Gamma (y+\frac{1}{k}){\left(k\lambda \right)}^{y}}{\Gamma (y+1)\Gamma (\frac{1}{k}){\left(1+k\lambda \right)}^{y+1/k}},\;y=1,2,\ldots .$$

District level characteristics along with the number of cholera cases in the districts were used in this analysis. Initially, we created bivariate models taking account of each variable in the model along with the number of cholera cases as the dependent variable. This was followed by multivariate analysis for those variable found to have association with the outcome at p<0.20 in the bivariate model. SAS version 9.4 was used to analyze the risk factors for cholera. We also calculated cumulative incidence rate over the six-year period and the coefficient of variation of the incidence rate based on the year-wise incidence rate of a district.

### Ethics

The study used secondary data aggregated at the district level, thus it did not require any ethical review board approval.

## Results

**Fig 1:**
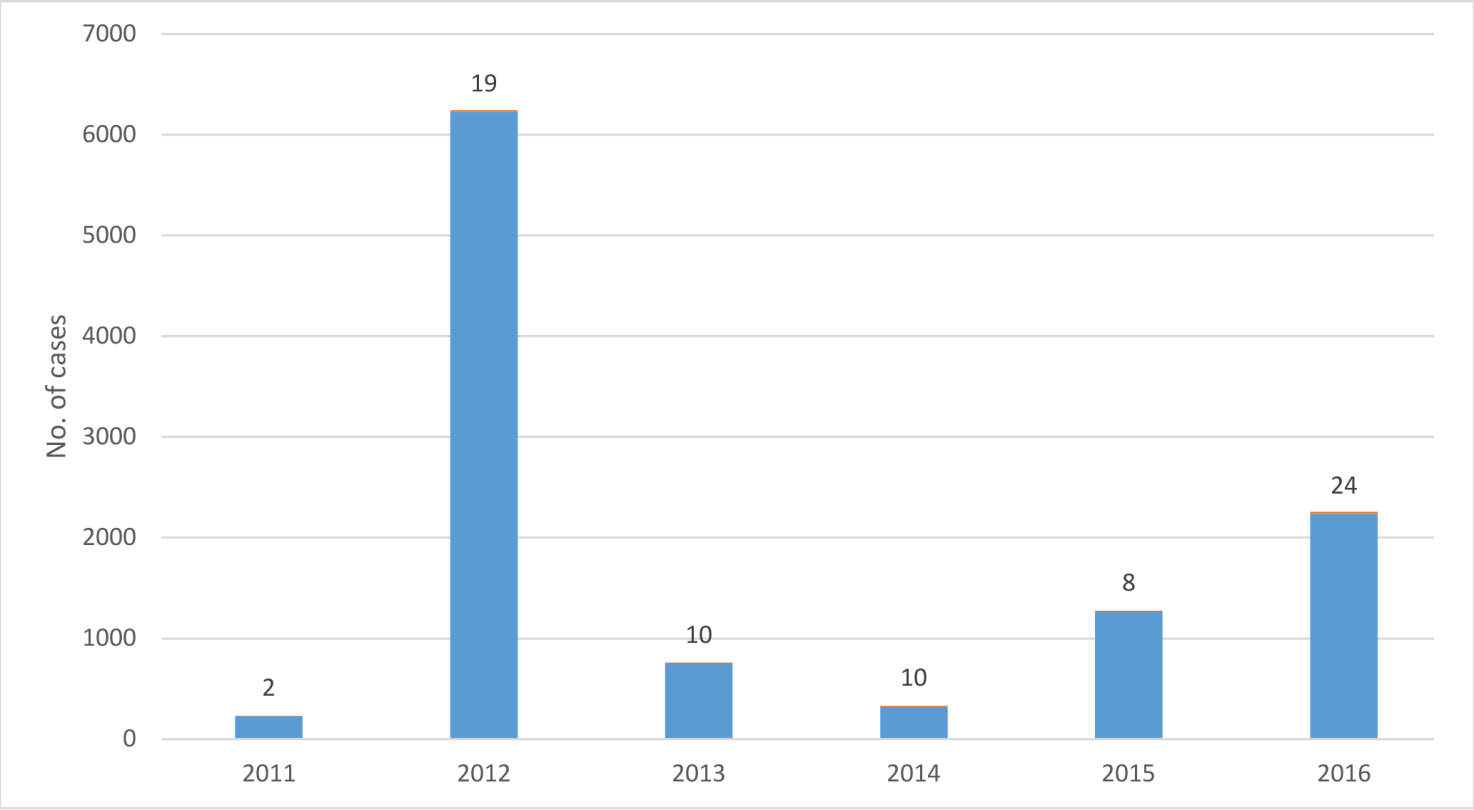
Distribution of cholera cases by year, 2011–2016, Uganda. No. of affected districts are shown on the top of bar.

A total of 11,030 cases of cholera were reported during 2011–2016. The highest number of cases were 6,226 in 2012 and the lowest were 229 in 2011 (Fig 1). In 37 of 112 districts (33%) cholera was reported in at least one of the study years. These districts made up 40% of the total population of Uganda. During the study period, the highest burden of cholera was in Nebbi district (2,320 cases) followed by Hoima (1,731 cases), and Buliisa (1,129 cases). The districts that had multiple outbreaks (at least two years) during study period had a population of about 7.8 millions.

**Fig 2:**
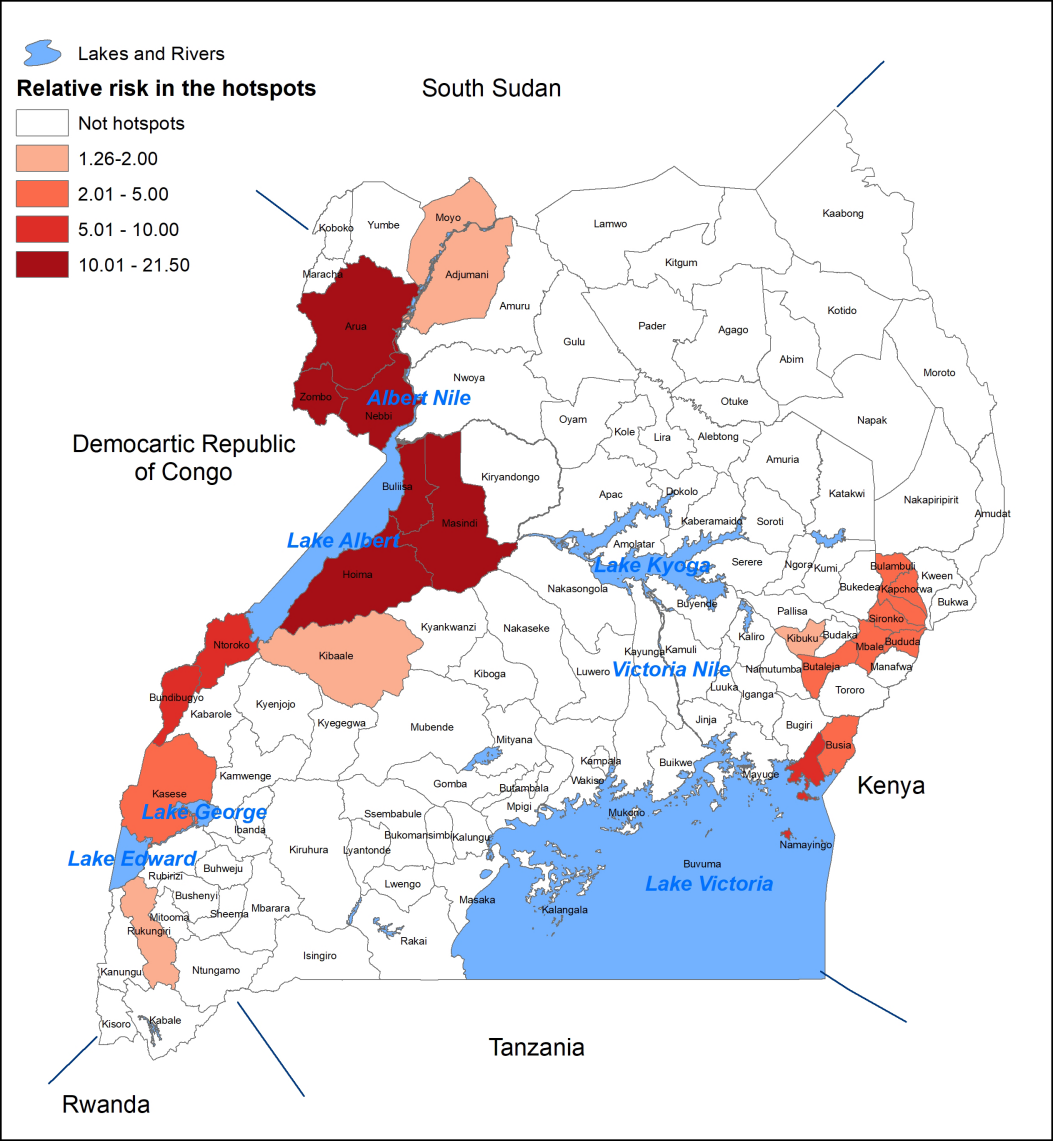
High risk districts (Hotspots) for Cholera in Uganda, 2011–2016.

The results of the SatScan for cluster detection based on the centers of the districts as the geographic coordinates yielded 16 significantly high-risk clusters of different sizes. Cholera hotspots were defined as districts whose center fell within a significantly high risk cluster or where significantly high risk cluster was completely superimposed onto a district. There were 22 districts whose centroids fell within the cluster detected by SatScan; these were defined as high risk districts. The risk of having cholera in these districts was 1.26 to 21.50 times compared to that elsewhere in the country (Table 1, Fig 2). About 7 million people live in these districts. Of the 22 districts, 13 of them (4.8 million people) are near the border of DRC and 9 of them (2.2 million people) are near the border of Kenya.

**Table 1:** Number of districts and total population in the hotspots of by risk groups.

Risk group	Relative risk	No. of districts	Total population
Low	1.26–2.00	5	1,666,078
Medium	2.01–5.00	8	2,484,056
High	5.01–10.00	3	506,834
Extremely High	10.01–21.50	6	2,396,213
Total	1.26–21.50	22	7,053,181

**Fig 3:**
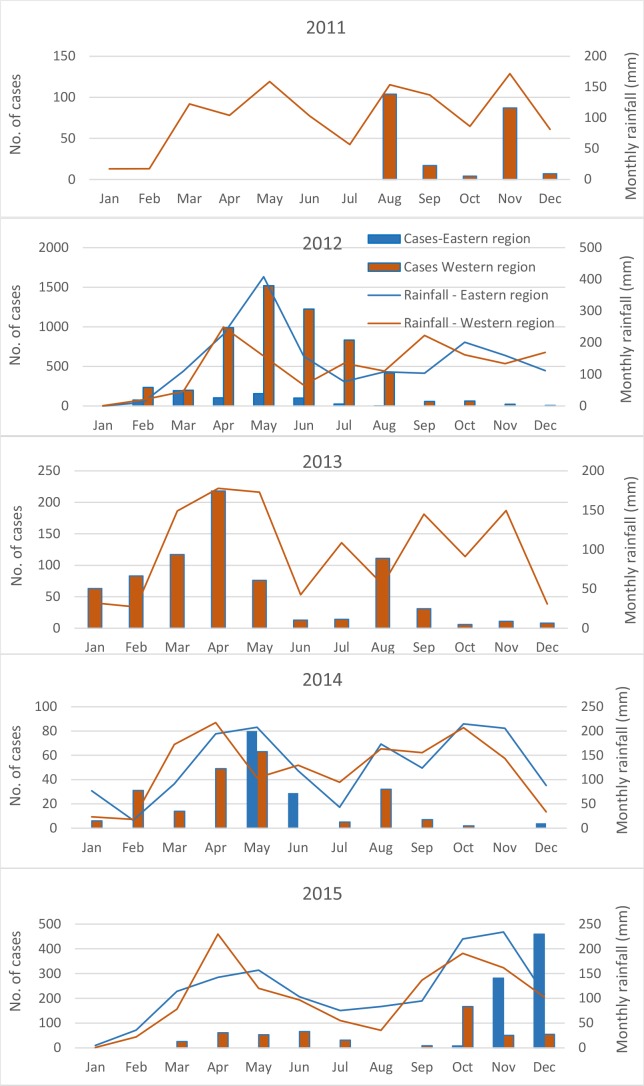
Month-wise distribution of the cholera cases and rainfall (mm) by year and by region (eastern vs. western) in Uganda, 2011–2015. Rainfall data of 2016 were not available, thus the graph of the year was not created.

**Fig 4:**
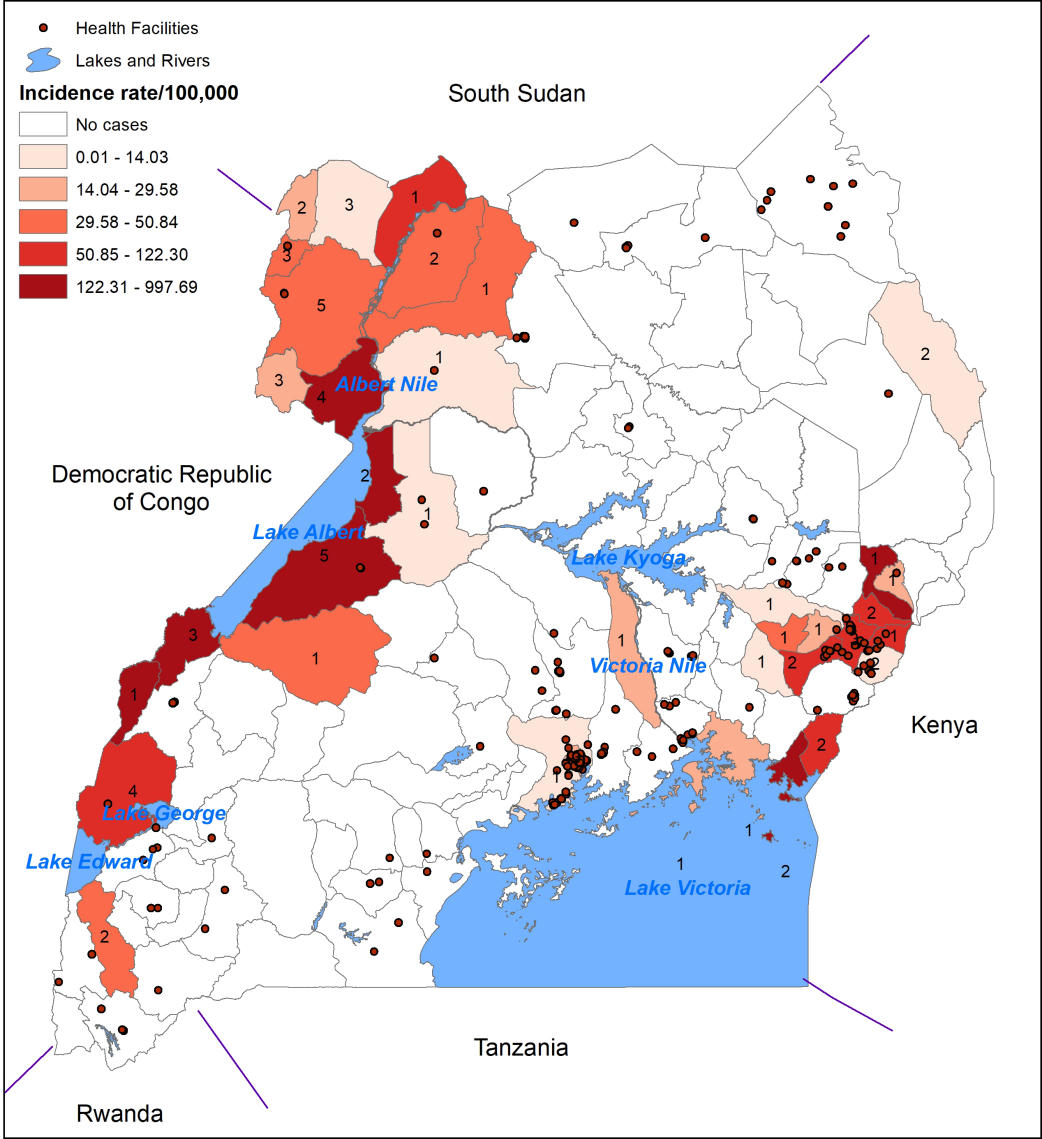
Spatial distribution of cumulative cholera incidence rates across six years (2011–2016) in Uganda.No. inside the district boundary states no. years of cholera occurrence during the study period.

We observed a distinct seasonal pattern of cholera between eastern and western region of Uganda. The seasonal pattern also varied by year. There was no cholera outbreak in the eastern region in 2011 and 2013. In 2012, the peak was between March and May in the eastern region, while in the western region the peak was between April and August (Fig 3). Apparently, there was only a little link with rainfall in a specific year and specific region (Fig 3). Fig 4 shows geographic distribution of the rates of cholera which varied in the study area, illustrating that cholera affected the people living near a lake or river. The coefficient of variations for the year-wise rates indicates that border districts experienced cholera more frequently than the inner part of the country (S2 Fig).

The basic statistics of the study variables show a high rate of improved water (82%) and sanitation (75%) coverage at the household level (Table 2). An average of 15% of the population lived in the urban areas. There were 300 hospitals/clinics for Uganda in the world health facility database, and these health facilities were heterogeneously distributed (visual perception) in space (Fig 4). The average distance to the nearest health facility, river of lake, and border of DRC or Kenya from the center of the district was 33, 47, and 91 kilometers, respectively. The average weighted cholera incidence rate (cumulative over the six year period) of the 1^st^ order of neighbors of a district was 28 per 100,000 persons. Average annual rainfall during 2011–2015 was 1324 mm.

**Table 2:** Descriptive statistics of the study variables by district (n = 112).

Variables	Mean	Median	Standard Deviation	Minimum	Maximum
Population -no.	309,238	239,704	252,204	54,293	1,997,418
Total cases in 2011–2016—no.	98	0	310	0	2320
Population density–per km^2^	276.86	179.07	730.16	7.01	7735.66
Urban population—%	15.30	12.74	12.91	1.87	100.00
Improved sanitation coverage—%	75.22	80.00	17.87	2.30	98.90
Improved handwashing coverage—%	33.06	33.05	15.21	0.00	86.00
Sanitation and hygiene benchmark *	51.92	52.00	13.91	15.00	90.00
Improved water sources—%	81.55	88.00	19.78	0.00	100.00
Distance from center of the district to nearest hospital—km	33.00	32.41	11.95	7.28	64.63
Distance from the center of the district to nearest lake or river—km	47.00	37.01	41.84	0	209.18
Distance from the center of the district to nearest border of DRC or Kenya—km	90.92	81.27	60.89	6.74	231.93
Weighted incidence rate of the 1^st^ order neighbor districts—per 100,000*	28.44	6.15	48.92	0	291.16
Average annual rainfall during 2011–2015 (mm)	1324.48	1440.70	194.34	939.06	1485.96

* Calculation of the benchmark score and weighted incidence rate of the 1^st^ order neighbor districts have been escribed in the text

The result of the bivariate analysis of the ZINB are presented in Table 3. The NB component of the model shows that higher incidence rate in a district was significantly associated (p < .05) with improved water sources, longer distance to the nearest heath facility, shorter distance to a lake or river, shorter distance to DRC or Kenya border, and a higher cholera incidence rate of the neighbor districts. On the other, although a significant relationship of population density was observed with reporting of cholera case by a district in the zero-inflated model, the point estimate does not indicate an increase or decrease of having cholera by the district. Likewise the NB model, distance to DRC or Kenya border and cholera incidence rate of the neighbor districts yielded a significant relationship (p < .05) with reporting of cholera cases in the zero-inflated model.

**Table 3:** Results of the zero-inflated negative binomial model in a bivariate analysis.

Variables	Negative binomial model			Zero-inflated model		
	Incidence Rate ratio*	95% CI	P-value	Odds ratio*	95% CI	P-value
Population density–per km^2^	1.00	1.00–1.00	0.1079	1.00	1.00–1.00	0.0088
Urban population—%	0.98	0.96–1.01	0.2042	1.01	0.98–1.04	0.3586
Improved sanitation coverage—%	0.99	0.95–1.04	0.7772	1.00	0.98–1.03	0.8170
Improved handwashing coverage—%	1.01	0.96–1.05	0.8074	1.00	0.98–1.03	0.7508
Sanitation and hygiene benchmark†	1.02	0.99–1.05	0.1349	1.00	0.97–1.03	0.9080
Improved water sources—%	1.03	1.00–1.05	0.0366	1.01	0.99–1.03	0.3766
Distance from center of the district to nearest hospital—km	1.03	1.00–1.07	0.0359	0.99	0.95–1.02	0.4052
Distance from the center of the district to nearest lake or river—km	0.98	0.98–0.99	0.0004	1.00	0.99–1.01	0.4195
Distance from the center of the district to nearest border of DRC or Kenya—km	0.98	0.97–0.99	< .0001	0.98	0.97–0.99	< .0001
Weighted incidence rate of the 1^st^ order neighbor districts—per 100,000†	1.01	1.00–1.02	0.0028	1.05	1.03–1.07	< .0001
Average annual rainfall during 2011–2015 (mm)	1.00	1.00–1.00	0.6943	1.00	1.00–1.00	0.0736

*Obtained by exponentiating the estimates obtained from the model

†Calculation of the benchmark score and weighted incidence rate of the 1^st^ order neighbor districts have been described in the text

We created multivariable model using the variables associated with the outcome at p<0.20. The results of the NB component of the multivariable model show that the distance to the nearest lake or river was significantly associated with the incidence of cholera (incidence rate ratio = 0.98, 95% CI: 0.97–0.99, p<0.01) (Table 4). This indicates that a one kilometer decrease in the distance to lake or river was associated with 2 percentage point of increase of cholera cases. Shorter distance to the DRC or Kenya border and higher cholera incidence of the neighbor districts were significantly associated (p < .05) with higher incidence rate of cholera in the district. On the other hand, the zero-inflated component of the multivariable model shows a one kilometer increase of distance to DRC or Kenya border was associated with 2 percentage point decrease in the odds of having cholera in the district. The zero-inflated model also shows that a one percentage point increase in the cholera incidence rate in the neighbor districts was associated with 3 percentage point increase in the odds of having cholera. Higher annual rainfall in the district was significantly associated (p = .03) with the risk of having cholera in the district in the zero-inflated model.

**Table 4:** Results of the zero-inflated negative binomial model in a multivariable analysis.

Variables	Negative binomial model			Zero-inflated model		
	Incidence Rate ratio*	95% CI	P-value	Odds ratio*	95% CI	P-value
Population density–per km^2^	1.00	1.00–1.00	0.4153	1.00	1.00–1.00	0.1123
Sanitation and hygiene benchmark†	1.01	0.98–1.04	0.5662	—‡	—	—
Improved water sources—%	0.99	0.97–1.02	0.6401	—‡	—	—
Distance from center of the district to nearest hospital—km	0.99	0.96–1.02	0.5354	—‡	—	—
Distance from the center of the district to the nearest lake or river—km	0.98	0.97–0.99	0.0008	—‡	—	—
Distance from the center of the district to nearest border of DRC or Kenya—km	0.99	0.98–1.00	0.0209	0.98	0.97–0.99	0.0142
Weighted incidence rate of the 1^st^ order neighbor districts—per 100,000†	1.02	1.01–1.03	0.0033	1.03	1.01–1.05	0.0002
Average annual rainfall during 2011–2015 (mm)	—‡	—	—	1.00	1.00–1.01	0.0338

*Obtained by exponentiating the estimates obtained from the model. The estimates cited herein are adjusted for all other variables in the model

†Calculation of the benchmark score and weighted incidence rate of the 1^st^ order neighbor districts have been described in the text

‡Variable not included in the model due to insignificant association with the outcome (p-value≥0.20) in the bivariate analysis

## Discussion

This results of our study show that most of the high risk districts for cholera were near the border with DRC and Kenya. These high risk districts with a population of about 7 million, make up about 20% of the population of Uganda. Those with a very high risk districts (relative risk was more than 10 compared to that elsewhere in the country) have a population of about 2.4 million (7% of the population of Uganda) from six districts. All of these districts are located near the DRC. Studies [36, 37] indicated that cross-border cholera outbreaks could be a major contributor to the recorded outbreaks in Uganda. Since a majority of the hotspot districts are near DRC or Kenya border, it suggests that a close collaboration with these countries would be an effective strategy for controlling cholera in that part of the world.

Our study also shows that proximity to a lake, specifically Lake Albert and Lake Victoria or to the Nile River, creates increased risk of cholera for the people in Uganda, as found in an earlier study [38]. Interestingly, rates of cholera are low near Lake Kyoga and along the Nile River as the water flows between Lake Kyoga and Lake Albert, but the rates are higher along the Nile River as the water flows north from Lake Albert. The finding in our study is consistent with earlier studies, which indicated that people are continuously being affected by cholera in lakeside areas [39]. Several other studies have also reported that Lake Albert and Lake Victoria could be a source of *Vibrio cholerae* [40, 41]. However, there is no definitive evidence to support the hypothesis. The link between high incidence of cholera and presence of lakes has also been noted in DRC [40, 42]. Bompangue et al [39] believe that in the absence of lakeside areas, the disease would have disappeared from the country. This may be true as we find cholera was less likely to create a threat to the people living far from the lakes. However, it is not clear if the lake is the major risk factor, or the behaviors or occupations of the people who live along the lake [43]. It should be noted that these lake areas also are borders with the neighboring countries with cholera; thus, it is difficult to separate the risk associated with the lake environment and the risk associated with cross border spread. The association between cholera outbreak and rainfall, as observed in our zero-inflated model, is consistent with earlier findings [39].

The seasonal pattern of cholera varied by year in Uganda suggesting exact timing of the outbreaks in the different regions of the country may not be predicted. A spike of cholera was observed in 2012, which was associated with a large outbreak in the African region that mostly affected six nations including Uganda (http://www.who.int/hac/crises/cholera_afro_22august2012.pdf?ua=1). In 2016, cholera showed a two distinct seasonal pattern between eastern and western region of the country. The peak season for cholera in eastern region started in March and continued until May, and the peak season in the western region started in June and lasted until August. Since Uganda experiences heavy rainfall from March to June, it seems that cholera outbreaks are associated with the rainfall in the country. Association of rainfall with the risk for cholera in Africa as well as in the neighboring country Kenya has already been observed [44, 45].

We did not find an association of water and sanitation with the risk for cholera in Uganda. This could be due to a reported high coverage of improved water sources and improved sanitation in the country. Also, the indicator used for the district which is an average for the district, may not represent the water and sanitation status of those who are vulnerable to cholera. With such high coverage for these WaSH indicators, one may want to reassess whether these indicators are providing an accurate reflection of the true situation for those at risk. The sustainability of water services in the small towns is also reported to be high (92%). This high coverage of access to safe water is largely due to back-up support for operations and maintenance which is being provided by regionally based Umbrella Organizations [33]. On the other hand, access to improved sanitation is reported to be high (85%) in the urban areas, and open defecation is reported to be rare in the both urban and rural areas of the country, except slums [33].

The strength of our study is that the cases of cholera were collected from a surveillance system which has been maintained systematically throughout the country and was done according to the WHO standard. Water and sanitation data were obtained from the report prepared by the Ministry of Water and Environment, Uganda, which are being updated on a yearly basis ensuring greater reliability of the data. The population data were obtained from a recent report prepared by the Uganda Bureau of Statistics [31] providing national population and housing census 2014, which is consistent with the period of our study. Additionally, since the data came from the national surveillance conducted by the Uganda Ministry of Health and was systematically executed throughout the country, we believe the burden of cholera across the districts are comparable.

The main limitation in our study is that cholera data were not population-based, which precluded calculating the absolute risk of the disease. Conducting a national level population-based disease surveillance in this setting is probably unrealistic. However, the data used in this study provided a basis for understanding relative burden of cholera across the districts in Uganda.

Our study identified cholera hotspots in Uganda that should be prioritized to accelerate reduction of cholera by implementation of targeted interventions. The intervention program includes provision of safe water, hygiene and sanitation, provision healthcare service and health education, and should be complemented with OCV and strong cross-border collaboration in outbreak prevention. The findings of our study could be used as a guide to strengthen the cholera control program in Uganda.

## Supporting information

S1 Fig Geographic characteristics of the study area. (TIF)

S2 Fig Coefficient of variation of the incidence rate of cholera in Uganda, 2011-2016.No. inside the district boundary states no. years of cholera occurrence during the study period. (TIF)

S1 Data Datafile.xlsx. (XLSX)
