# Supplementary figures and images for: Identifying cholera "hotspots" in Uganda: An analysis of cholera surveillance data from 2011 to 2016
Source: PLoS Negl Trop Dis. 2017 Dec 28;11(12):e0006118. doi: 10.1371/journal.pntd.0006118 (PMC5746206; doi:10.1371/journal.pntd.0006118)

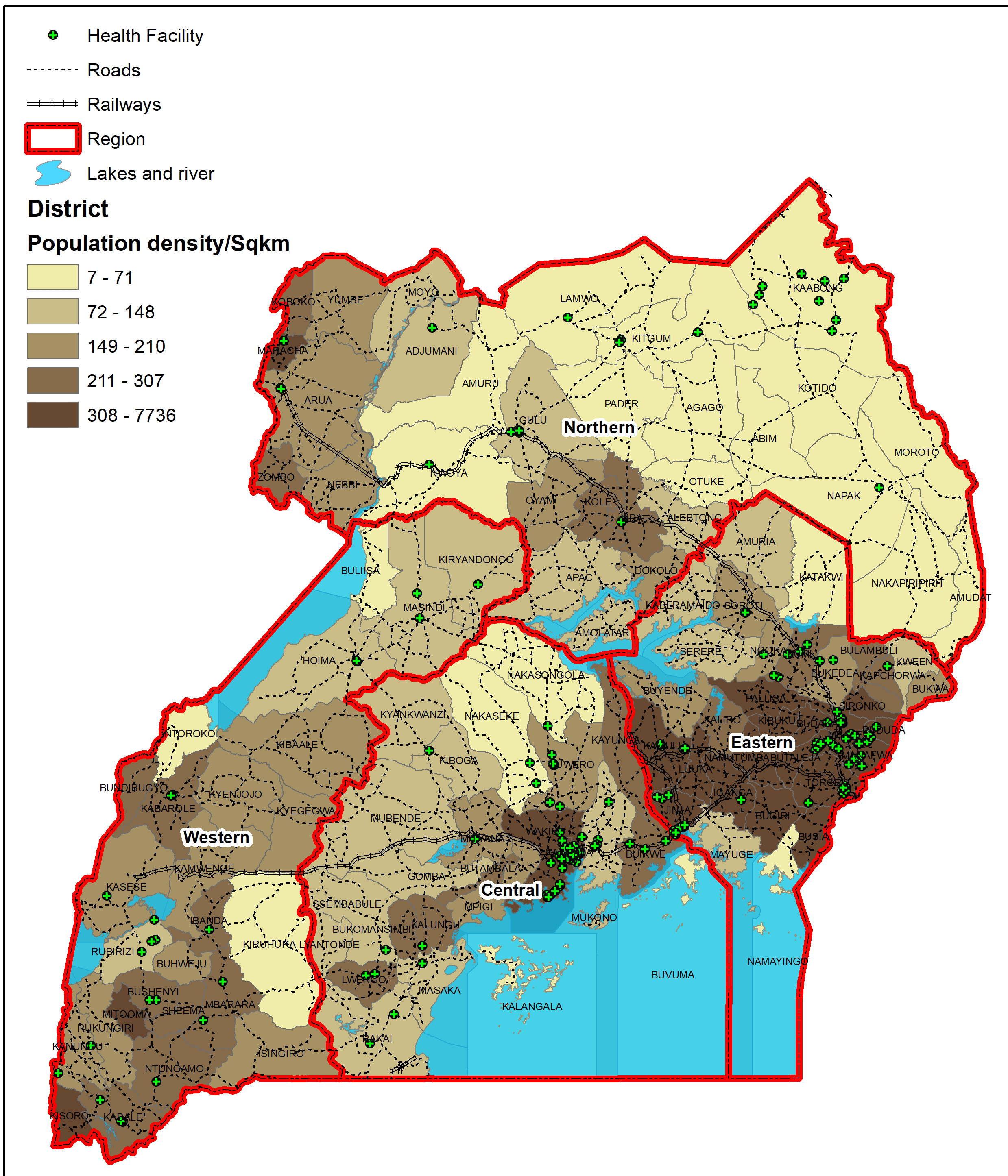

Supplement: S1 Fig — (TIF) [file pntd.0006118.s001.tif]

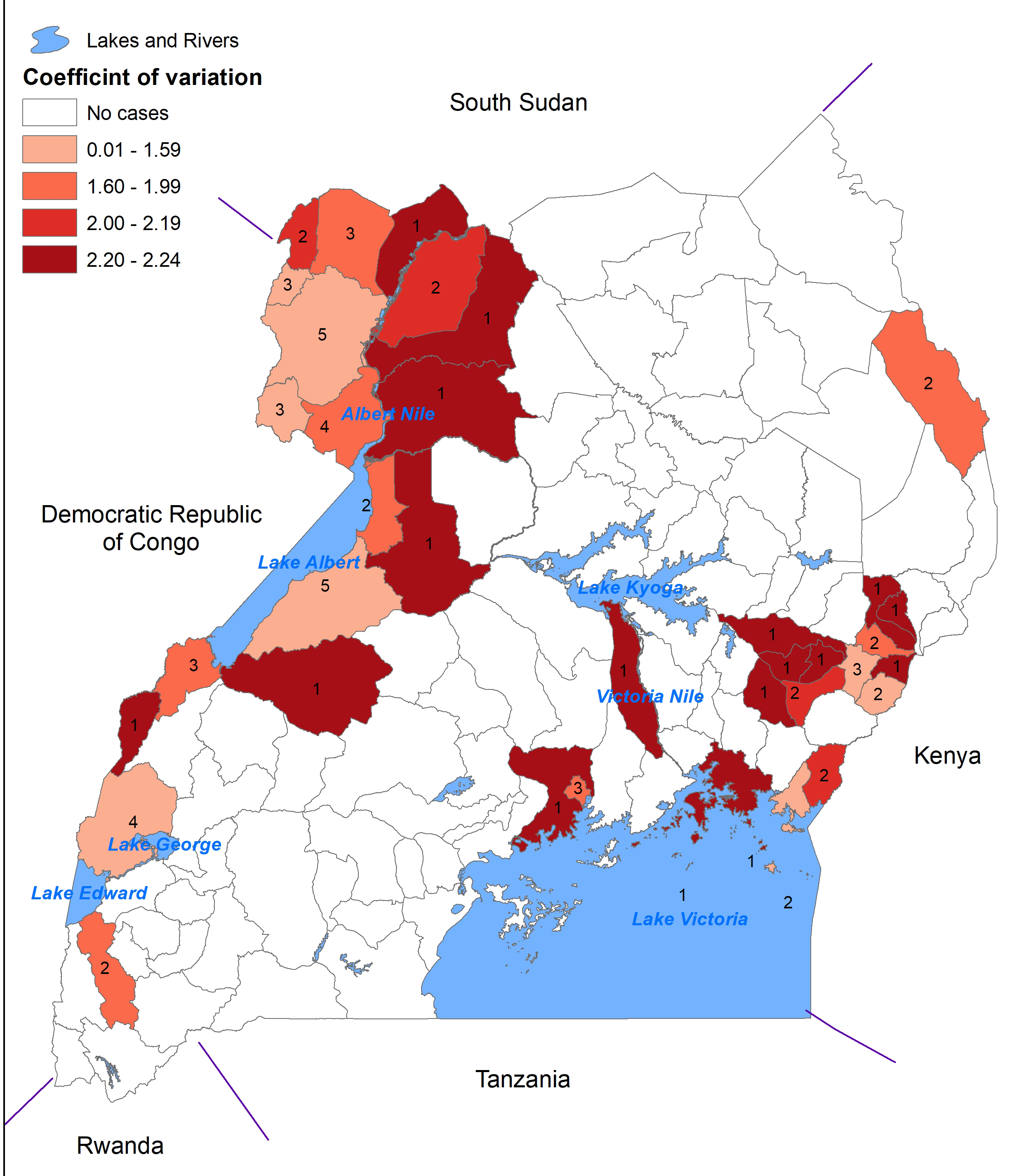

Supplement: S2 Fig — (TIF) [file pntd.0006118.s002.tif]
